# Neutrophil Extracellular Traps and Cardiovascular Diseases: An Update

**DOI:** 10.3390/cells9010231

**Published:** 2020-01-17

**Authors:** Aldo Bonaventura, Alessandra Vecchié, Antonio Abbate, Fabrizio Montecucco

**Affiliations:** 1 Pauley Heart Center, Division of Cardiology, Department of Internal Medicine, Virginia Commonwealth University, 1200 E Marshall St, Richmond, VA 23298, USA; alessandra.vecchie@vcuhealth.org (A.V.); antonio.abbate@vcuhealth.org (A.A.); 2 First Clinic of Internal Medicine, Department of Internal Medicine, University of Genoa, viale Benedetto XV 6, 16132 Genoa, Italy; 3 First Clinic of Internal Medicine, Department of Internal Medicine and Centre of Excellence for Biomedical Research (CEBR), University of Genoa, viale Benedetto XV 6, 16132 Genoa, Italy; fabrizio.montecucco@unige.it; 4 IRCCS Ospedale Policlinico San Martino Genova—Italian Cardiovascular Network, Largo R. Benzi 10, 16132 Genoa, Italy

**Keywords:** NETs, neutrophils, NLRP3 inflammasome, IL-1β, cardiovascular disease, inflammation, diabetes, obesity

## Abstract

Neutrophil extracellular traps (NETs) are formed by decondensed chromatin, histones, and neutrophil granular proteins and have a role in entrapping microbial pathogens. NETs, however, have pro-thrombotic properties by stimulating fibrin deposition, and increased NET levels correlate with larger infarct size and predict major adverse cardiovascular (CV) events. NETs have been involved also in the pathogenesis of diabetes, as high glucose levels were found to induce NETosis. Accordingly, NETs have been described as drivers of diabetic complications, such as diabetic wound and diabetic retinopathy. Inflammasomes are macromolecular structures involved in the release of pro-inflammatory mediators, such as interleukin-1, which is a key mediator in CV diseases. A crosstalk between the inflammasome and NETs is known for some rheumatologic diseases, while this link is still under investigation and not completely understood in CV diseases. In this review, we summarized the most recent updates about the role of NETs in acute myocardial infarction and metabolic diseases and provided an overview on the relationship between NET and inflammasome activities in rheumatologic diseases, speculating a possible link between these two entities also in CV diseases.

## 1. Introduction

**Figure 1:**
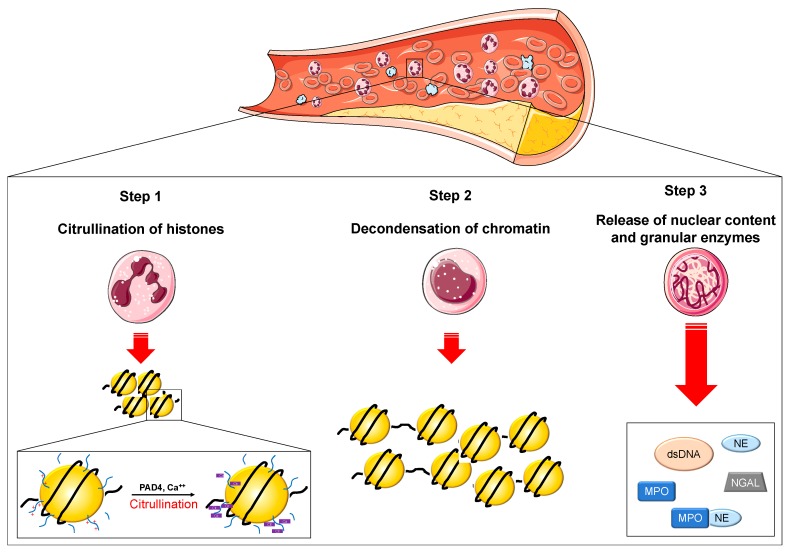
Overview of NETosis. NETosis occurs through the release of neutrophil extracellular traps (NETs) and represents an additional mechanism of defense. The cascade of events leading to NETosis include the histone tail citrullination of positively charged arginine residues mediated by the calcium-dependent PAD4 (Step 1), that leads to chromatin decondensation (Step 2). After this, the nuclear envelope destroys, the granule content enters the nucleus followed by the release of the nuclear material along with granular enzymes (Step 3). Legend. Ca++: calcium. Cit: citrullinated histone tail. dsDNA: double-stranded deoxyribonucleic acid. PAD4: peptidylarginine deiminase type 4. MPO: myeloperoxidase. NE: neutrophil elastase. NGAL: neutrophil gelatinase-associated lipocalin.

Neutrophils are the most abundant effector cells of the innate immune system [1]. Apart from their defensive role against infections, neutrophils have acquired a distinct role in the pathophysiology of many cardiovascular (CV) diseases [2]. The most important mechanism for their phagocytic and antimicrobial activity is the release of granular products (i.e., metalloproteinase [MMP]-8 and -9, myeloperoxidase [MPO], neutrophil gelatinase-associated lipocalin [NGAL], and neutrophil elastase [NE]) [2,3]. More recently, neutrophil extracellular traps (NETs) have been recognized as an additional mechanism of defense through a process called NETosis [4,5]. NETs are made up of chromatin decorated with histones, proteases, and granular proteins through which neutrophils can block and catch up invading microorganisms [6,7] (Figure 1). The presence of granular proteins is an essential requirement for NET formation since both NE knockout mice and MPO-deficient patients were found to produce a lesser amount of NETs [8,9]. Indeed, in experimental studies, mice unable to activate NETosis showed a higher susceptibility to infectious diseases [10]. NETs, however, have been recognized as important drivers also in the pathophysiology of CV diseases [2,11,12].

Inflammasomes are intracellular, macromolecular complexes that sense dangers and trigger a local or systemic inflammatory response through the release of cytokines belonging to the interleukin (IL)-1 family [13]. In particular, the NACHT, LRR, and PYD domain-containing protein 3 (NLRP3) inflammasome is thought to act as a central player in the setting of acute myocardial infarction (AMI) and heart failure [13]. A crosstalk between the inflammasome and NETs was described in different settings, with NETs able to prime macrophages to produce IL-1 through the NLRP3 inflammasome, thus amplifying the inflammatory response [14], although this interplay is less understood in CV diseases.

In this review, we aim to summarize the most recent findings concerning the role of NETs in AMI and metabolic diseases (particularly diabetes and obesity). In addition, we provide some information on the relationship between NET and inflammasome activities in rheumatologic diseases, speculating a possible link between these two entities in CV diseases.

## 2. Review Criteria

This narrative review is based on original articles and reviews published over the last years and retrieved through PubMed using the following search terms (or combination of terms): NETs, neutrophils, NE, MPO, inflammation, NLRP3 inflammasome, acute myocardial infarction, ST elevation myocardial infarction, obesity, type 1 diabetes, type 2 diabetes, and outcomes. Only English-language papers were included. Additional papers identified from the reference list of the retrieved articles were also considered.

## 3. NETs in Acute Myocardial Infarction

Acute myocardial infarction (AMI) is triggered in most cases by the erosion/rupture of a coronary atherosclerotic plaque, followed by the formation of a thrombus occluding the artery [15]. Although monocytes and macrophages are known to play an essential role, in recent years neutrophils have also been described to be involved in atherothrombosis [16,17], as demonstrated by their accumulation in coronary thrombi [18] and their predictive role in acute coronary events [19,20]. As well, neutrophils and NETs were described in animal models of ischemia/reperfusion injury (IRI) along with the beneficial effect of DNase in mitigating the IRI and the no-reflow phenomenon [21].

**Figure 2:**
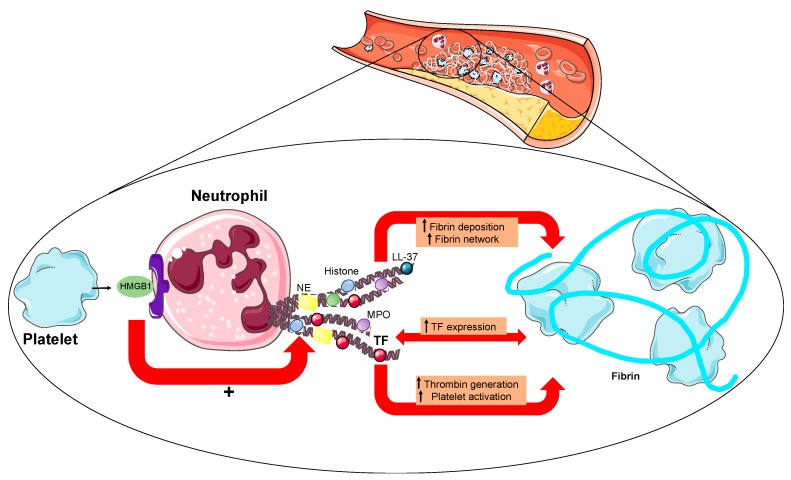
The interplay between NETs and platelets. NETs promote thrombosis by favoring fibrin deposition. Recently, a notion has been added by showing that NETs can express tissue factor further triggering thrombin generation and platelet activation and finally increasing the thrombogenic potential of NETs. This was reported especially at the site of plaque rupture during acute myocardial infarction when platelets and neutrophils interact with each other. In this view, activated platelets present HMGB1 to neutrophils and stimulate them to form NETs. Legend. HMGB1: high-mobility group box 1. NE: neutrophil elastase. MPO: myeloperoxidase. TF: tissue factor.

NETs play a central role in thrombosis by promoting fibrin deposition and formation of fibrin networks [22]. Following the interaction between platelets and neutrophils at the site of plaque rupture during STEMI, NETs were recognized to express functional tissue factor and to induce platelet activation and further thrombin generation, thus increasing the thrombogenic potential of NETs [23] (Figure 2). Importantly, the integrity of the deoxyribonucleic acid (DNA) scaffold was proved as a necessary condition for the tissue factor to be expressed on NETs from infarct-related coronary arteries [23]. Despite the presence of NETs in developing thrombi, human intact NETs did not show any pro-coagulant property in vitro in contrast to single histones able to induce thrombin generation in a platelet-dependent way [24]. This probably depends on the neutralization of the negative charge of DNA on the NET surface. Additionally, it is now clear that activated platelets can present high-mobility group box 1 (HMGB1) to neutrophils and stimulate these cells to form NETs [25] (Figure 2). More recently, a concept emerged following the discovery that toll-like receptor (TLR)-2 stimulation along with NETs trapped within the fibrin strands might have a role in plaque erosion through an increase in endoplasmic reticulum (ER) inducing ER stress and apoptosis [26].

A central role for NETs in coronary artery disease (CAD) has been proposed and main studies investigating this relationship are summarized in Table 1. For example, NETs were retrieved at a higher extent in older thrombi showing lytic changes compared to fresh ones, but never in organized thrombi, thus suggesting that NET formation occurs early in the thrombus dissolution process [27]. In another study, coronary thrombi were confirmed to contain a large amount of NETs, which are deemed as a scaffold for platelets, red blood cells, and fibrin [28].

Nucleosomes (DNA-histone complexes) and double-stranded DNA (dsDNA)—key components of NETs proposed as sensitive biomarkers for CV events—were found at increased levels at the culprit site and correlated with the coronary thrombus NET burden. Interestingly, the latter negatively correlated with ST resolution, but was positively associated with the infarct size expressed both as area under the curve of creatine phosphokinase isoform muscle brain (CK-MB) and through cardiac magnetic resonance (CMR) assessment [28]. These data underline the detrimental role played by NETs at the culprit site through the stimulation of thrombosis and inflammation into the infarcted myocardium. Additionally, the activity of DNase at the culprit site was negatively correlated with the coronary NET burden, the area at risk, ST resolution, and infarct size measured through CMR [28]. Hence, the balance between NET burden and endogenous DNase activity might be responsible for different outcomes and eventually represent a field of research for targeted therapies. This is also true for other surrogate markers of NET burden (dsDNA and citrullinated histone H3 [citH3]), whose concentration was increased at the culprit site [32]. Interestingly, in patients evaluated through transthoracic echocardiography after 24 ± 8 months from AMI, dsDNA at the culprit site positively correlated with the wall motion score index (the higher the index, the worse the left ventricular function) [32]. Another study pointed out the prognostic role of NETs. In a cohort of patients with recent STEMI, higher levels of coronary dsDNA were found to independently predict in-hospital major adverse CV events (MACEs) [33]. These results, however, confirmed those previously reported by Borissoff et al., who in 2013 described for the first time a positive association between high levels of circulating dsDNA, nucleosomes, and MPO-DNA complexes and the occurrence of MACEs [29].

Some studies followed the time course of NETs at different time points. In a cohort of 30 patients with STEMI and stable angina undergoing successful PCI [31], a progressive decrease of dsDNA in all patients following PCI was reported across 14 days, although STEMI patients exhibited higher levels throughout the observation time. Differently, nucleosomes were found to peak after 12 h from PCI in STEMI patients and then to progressively reduce until day 7 with a slight increase at day 14, remaining higher in patients with stable angina [31]. In another study, both dsDNA and MPO-DNA concentrations were higher in the acute phase than after three months and correlated with the total leukocyte count at the time of the admission and to each other only in the acute phase. Interestingly, the acute glucose load provided by the oral glucose tolerance test performed three months after the STEMI resulted in an increased gene expression of peptidylarginine deiminase 4 (PAD4), but it was not paralleled by an increase in dsDNA and/or MPO-DNA levels [34]. Based on the available literature [37], the authors concluded that this may be probably due to a delayed release of NETs or to the fact that NETosis might be triggered by acute changes in plasma glucose in the stable setting (i.e., three months) following an AMI.

NETs are likely to behave in a similar manner also in stable CAD. In a large study including >1000 patients previously enrolled in the ASCET (ASpirin non-responsiveness and Clinical Endpoints Trial) [38], dsDNA and MPO-DNA significantly correlated with neutrophil count, but only dsDNA levels were strongly associated with prothrombin fragment 1 and 2 and D-dimer, two in vivo markers of thrombin generation and fibrin turnover hypercoagulability. These findings further underpinned how NETs exert a negative impact beyond their prothrombotic potential [38] and confirmed previous findings by Borissoff et al. [29]. When analyzing the outcomes (including a composite of unstable angina, non-hemorrhagic stroke, AMI, or all-cause mortality), patients experiencing an event presented with significantly higher dsDNA levels as compared to patients without events. A trend toward an increased number of endpoints across quartiles was found, especially for quartiles from 2nd to 4th. Additionally, increased dsDNA levels in 2nd–4th quartiles were associated with a 2-fold increased risk of experiencing a composite of unstable angina, non-hemorrhagic stroke, AMI, or all-cause mortality, independently of treatment allocation and markers of hypercoagulability [38].

## 4. NETs in Diabetes

Diabetes is characterized by a low-grade inflammation [39] explaining the typical complications of the disease through endothelial dysfunction, hyperreactivity of platelets, and elevated levels of pro-coagulant mediators [40,41]. Hyperglycemic conditions were reported to limit lipopolysaccharide (LPS)-induced neutrophil degranulation and the following release of granular proteins, i.e., MPO and NE [42,43]. In recent years, a progressive number of evidences shed light on the role played by neutrophils in the pathophysiology of diabetes (both type 1 diabetes [T1D] and type 2 diabetes [T2D]) [44,45,46,47]. Additionally, the expression of PAD4 in neutrophils is elevated in patients with T1D and T2D, thus explaining their special attitude to produce NETs [48].

Neutrophils were recognized to infiltrate the pancreas of subjects with T1D [49] and to have a role in the onset and the progression of T1D [50]. Neutrophils might accumulate at all disease stages, including pre-symptomatic subjects with positive autoantibodies [51]. Through the colocalization between dsDNA and MPO, it emerged that pancreas-residing neutrophils were able to form NETs, as confirmed by the decoration of decondensed DNA with citrullinated histones [51]. Indeed, a reduction in the neutrophil count in patients with T1D at onset was paralleled by a marked increase in NE and proteinase 3 (PR3) levels and activity, that in turn were associated with an increased NETosis [49,50,52,53]. It is then likely that the reduced number of circulating neutrophils might be due to an increased NET production, which is also responsible for the augmented levels of NE and PR3 in the bloodstream.

Additional studies are available describing the role played by NETs in T2D, but they appear controversial. Although high glucose levels were described to induce NETosis and NET-related products both in vitro and in vivo in a dose-dependent fashion [54], both spontaneously and when induced by phorbol 12-myristate 13-acetate (PMA) [55], a deeper investigation provided somewhat different results. In fact, Joshi et al. reported that in vitro NET formation was impaired at progressively increasing glucose concentrations, being delayed and resulting in short-lived and unstable NETs [56]. This was later confirmed when neutrophils from T2D subjects were stimulated for 3 h with LPS. In patients with T2D, a pre-activated condition was observed, i.e., NETting neutrophils without any external stimulus, only in presence of normal glucose concentration (5.5 mM). This was probably due to the incapacity of neutrophils to respond to other stimuli (e.g., PMA) when already exposed to hyperglycemia [56]. An explanation for these different results in pretty similar conditions may be the different time of incubation (4 h vs. 24 h, respectively) affecting the process of NET formation. Hence, diabetes represents a trigger for the constitutive NET activation, but the continuous stimulus provided by higher glucose concentrations is likely to detrimentally impact on the ability of neutrophils to produce fully working NETs. This aspect, therefore, deserves further investigation in the future in order to better clarify these mechanisms.

Neutrophils from T2D patients were shown to produce a large amount of IL-6, which in turn induced NET formation in an autocrine fashion at the same extent of LPS, except when neutrophils were cultured under high glucose conditions (30 mM) [56]. This latter finding was confirmed in a small cohort of T2D patients followed for one year from the time of the diagnosis and treated with metformin 500–2500 mg/daily [57]. Indeed, neutrophils from T2D patients produced a larger amount of NETs compared to healthy controls, but not when stimulated with PMA [57]. Additionally, NET formation, nucleosome, and NE-dsDNA complexes were still present in the plasma of patients after 6 months of metformin treatment, while all neutrophil functional responses returned to normal values after 12 months with no change in neutrophil count across this period [57]. This finding about metformin was later investigated by Menegazzo et al. confirming that metformin reduced NE, PR3, histones, and dsDNA levels [58]. Indeed, metformin blocked in vitro pathologic changes in nuclear dynamics and DNA release provoking a blunted NETosis in response to classical NET stimuli [58]. As NET formation is likely to be associated with glucose-stimulated reactive oxygen species (ROS) production, it is possible that ROS have a direct effect on NET production. In fact, diphenyleneiodonium and apocynin, two inhibitors of NADPH oxidase, reduce NET formation in neutrophils exposed to high glucose stimulation [59]. Accordingly, the inhibition of NADPH oxidase markedly decreased the release of extracellular DNA compared to high glucose condition, thus suggesting that glucose-induced NET production might be dependent on NADPH oxidase [59].

NETs were described to be implicated in diabetic complications. Indeed, a poor outcome for diabetic wounds in subjects with T1D and T2D was recorded [48,60]. In a case-control association study, dsDNA-histone complex and NE levels were significantly increased in patients with diabetic retinopathy compared with those without [55]. As a further proof of it, Wang et al. reported that an increased NET formation was observed in the serum of T2D patients with diabetic retinopathy irrespective of the stage of the disease, with NE driving this trend [59].

## 5. NETs in Obesity

Obesity is considered an inflammatory disease since adipose tissue dysfunction is responsible for an impairment in adipocytokine production [61]. In addition, inflammatory cells can infiltrate the adipose tissue [62] and release pro-inflammatory mediators [63,64]. NETs were shown to have a role in the obesity-related inflammation both in pre-clinical and clinical studies.

In an experimental mouse model of high-fat, high-sucrose diet, the immunostaining for cathelicidin-related antimicrobial peptide (CRAMP), a surrogate marker of NETting neutrophils, was markedly more positive compared to control lean mice and reduced after treatment with Cl-amidine, a PAD4 inhibitor, or DNase [65]. Additionally, the effect of NETs in mediating endothelial dysfunction in obese mice was studied with Cl-amidine administered for 2 weeks or DNase for 8 days, after 8 and 9 weeks of high fat, respectively. Blocking NETs is beneficial for the recovery from the endothelial dysfunction provoked by the high-fat diet [65]. The main explanation of the role of NETs in obesity-induced endothelial dysfunction might be an abnormal production of MPO. MPO, in fact, increases the production of ROS, which in turn oxidize the endothelial-derived nitric oxide production. Different results were reported by Braster et al. in another model of high-fat diet, in which the presence of NETting neutrophils in the adipose tissue was confirmed at a higher extent in obese than in lean mice [66]. Despite the administration of Cl-amidine for 10 weeks since the beginning of the high-fat diet, no beneficial effect in terms of improved metabolic parameters was found [66]. This is likely to suggest that an early blockade of NETs might not be effective in blunting NET-related effects probably due to the presence of a kind of ‘escape’ mechanism.

Recently, in a cohort of patients with morbid obesity who underwent sleeve gastrectomy, a higher amount of MPO-DNA complexes compared to healthy controls was found [67]. Levels of MPO-DNA complexes positively correlated with body weight, body mass index, waist and hip circumference, and glyco-metabolic profile. One year after the surgical intervention, MPO-DNA complexes did not show any absolute change compared to baseline. In some patients, however, MPO-DNA complexes were reduced, whereas in some others increased [67], thus suggesting that the mere weight loss may not modify neutrophil activation. Interestingly, the sub-group with reduced MPO-DNA complexes after sleeve gastrectomy presented with a reduced body weight and BMI and an improved glycemic status. On the contrary, those with persisting high levels of MPO-DNA complexes after surgery had a history of stroke and thromboembolism and therefore may represent a high CV risk population [67].

## 6. NETs and the Inflammasome

Depending on different sensor components, various inflammasomes can oligomerize [68,69] and the activation of the caspase-1 takes place [70]. The common, final stage of this process is the proteolytic cleavage of pro-IL-1β and pro-IL-18 to their active forms. The NACHT, LRR, and PYD domain-containing protein 3 (NLRP3) inflammasome was largely studied in the CV field, as confirmed by their role in atherosclerosis, acute myocardial infarction, heart failure, and pericarditis [71,72,73,74,75,76]. Of note, the role of the NLRP3 inflammasome and IL-1β is well established in many rheumatic diseases, too [77].

**Figure 3:**
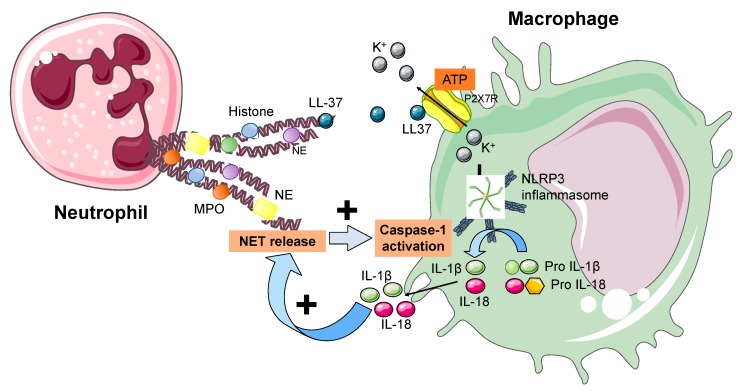
Interactions between neutrophils and macrophages. The NLRP3 inflammasome is an intracellular macromolecular structure recognizing danger signals and activating an inflammatory response, especially by the release of IL-1β and IL-18. In recent works, NETs have been described to play a central role in activating the NLRP3 inflammasome in macrophages. This happens through LL-37 triggering potassium efflux from the cell via the activation of the P2X7R. Additionally, IL-18 may stimulate NET release and increase caspase-1 activation in macrophages in a feed-forward loop. Accordingly, NETs trigger IL-1β and IL-18 synthesis in macrophages, that promote NET formation in neutrophils. Legend. IL: interleukin. MPO: myeloperoxidase. NET: neutrophil extracellular trap. NLRP3: NACHT, LRR, and PYD domain-containing protein 3. P2X7R: P2X purinoreceptor 7.

Cathelicidin LL-37 is an antimicrobial peptide released within NETs and was previously recognized to induce IL-1β from monocytes following the activation of the P2X purinoreceptor 7 (P2X7R) [78], that mediates the potassium efflux during inflammasome activation (Figure 3). Kahlenberg et al. reported some interesting findings on the role played by NETs in triggering the NLRP3 inflammasome activation in macrophages of patients with systemic lupus erythematosus (SLE) [79]. Indeed, they found a positive correlation between the concentration of LL-37 within NETs and their ability to activate the NLRP3 inflammasome and release IL-1β and IL-18 through caspase-1. As well, LL-37 played a major role in potassium efflux through the P2X7R activation. A central role for IL-18 was shown, which effectively stimulated NET release at the same extent of other known NETosis stimuli (i.e., PMA and LPS) [79]. Additionally, IL-18-stimulated NET release significantly increased caspase-1 activation in primed macrophages compared to IL-18 alone. This might suggest a feed-forward loop through which NETs increase the synthesis of IL-1β and IL-18 in macrophages, that in turn can stimulate NET formation in neutrophils (Figure 3). Similarly to what happens in SLE, the pivotal role of NETs was demonstrated in acute gout, too [80]. As well, patients with adult-onset Still’s disease (AOSD) presented with higher levels of circulating NETs compared to controls, which contributed to the NLRP3 inflammasome and macrophage activation, finally increasing the release of pro-inflammatory cytokines [81]. An interplay between NETs and the inflammasome has been described also in severe asthma [82]. Among patients included in the Severe Asthma Research Program (SARP)-3, increased caspase-1 concentrations were measured in patients with high levels of NETs, thus suggesting the inflammasome activation. This may suggest that patients with severe asthma have a marked neutrophil activation in their airways, as proved by the increased concentrations of NETs, the latter being able to further trigger the inflammasome in monocytes or macrophages and release IL-1β [82,83].

Caspases are important in pyroptosis, a regulated cellular death depending on the activation of the inflammasome [84,85]. Typical substrates are IL-1β and IL-18. Recently, gasdermin D (GSDMD) has been identified as an important pyroptotic effector activated by caspase-11 and less often by caspase-1 [86,87]. Caspases are responsible for the cleavage of the Asp275 and Asp276 residues on GSDMD, that generates an N-terminal GSDMD product, namely GSDMD-NT or GSDMD-p30. GSDMD-NT is responsible for the pores through the cytoplasmic membrane [88,89] leading to osmotic swelling and membrane rupture. Pyroptosis can, however, be also activated in a non-canonical fashion through caspase-11 (caspase-4/5 in human cells) activity following LPS stimulation [87].

Recently, Sollberger et al. investigated the role of GSDMD as a common effector of both pyroptosis and NETosis [90]. In an in vitro experiment, GSDMD was cleaved during NET formation and localized to the plasma membrane of neutrophils. Additionally, some neutrophil proteases (NE, PR3, and cathepsin G) were found to induce GSDMD cleavage at different sites leading to lysis-inducing fragments. The authors then concluded that GSDMD may represent a key player in pro-inflammatory cell death through two functions [90]. First of all, NE and GSDMD look like to be involved in a feed-forward loop, in which GSDMD and NE help each other for their own activation. Once GSDMD activates, it is able to form pores in the membrane of granules favoring NE release into the cytoplasm toward the nucleus, where it gets processed with histones and increases nuclear expansion [91]. As a second function, following NET formation, GSDMD forms pores in the cell membrane allowing NET release. Along with these interesting findings and the knowledge that neutrophils appear to be resistant to caspase-1-dependent pyroptosis, neutrophils were recently described to release NETs in a GSDMD-dependent manner following the non-canonical inflammasome signaling [92]. Indeed, pyroptosis was previously described to depend on the proteolytic cleavage of GSDMD by caspase 11 in neutrophils [86,87].

The role of the NLRP3 inflammasome in AMI was largely investigated in recent years [93,94,95]. Actually, we now know that the NLRP3 inflammasome activation is important in the determination of the infarct size (mainly through pyroptosis) [96,97] and in the so-called “wavefront of reperfusion injury,” through which the infarct size expands in the 3–6 h after reperfusion through an increasing activation of the NLRP3 inflammasome [94]. For a more in-depth discussion on the role of the NLRP3 inflammasome, readers are referred elsewhere [13,75,95]. Therefore, the link between NETs and the NLRP3 inflammasome in the pathophysiology of AMI is still under investigation and not completely understood yet. Cholesterol crystals are recognized as important drivers in atherosclerosis [98], as supported by studies in mice with defective cholesterol efflux [99,100]. Additionally, the excess of cholesterol crystals is uptaken by lysosomes, whose membrane may be damaged and then activate the NLRP3 inflammasomes [99]. Besides, cholesterol crystals were found to prime the NLRP3 inflammasome through the production of NETs [101].

With regard to diabetes, very few data are available evaluating the relationship between NETs and the inflammasome, although data from the CANTOS (Canakinumab Anti-Inflammatory Thrombosis Outcomes Study) trial did not show any effectiveness of IL-1β blocking in reducing incident diabetes [102]. Future studies unraveling this interplay may be beneficial in developing targeted therapies for patients developing diabetes.

## 7. Conclusions and Future Perspectives

NETs primarily represent an essential barrier in response to inflammatory stimuli provided by a wealth of pathogens. An excessive production of NETs may, however, end in chronic inflammation, as occurs in CV diseases, where NETs play an important role. Additionally, NETs can trigger other cells, such as monocytes and macrophages, to release IL-1β through the NLRP3 inflammasome, which in turn is responsible for the persistence of a pro-inflammatory milieu. In vitro studies reported that proteases on NETs can adjust cytokine levels either by destroying or activating them, thus blunting or favoring inflammation [103,104,105]. Therefore, NETs are likely to behave as a double-edged sword in the innate immune system. An exaggerated NET formation is responsible for different diseases, both infectious and non-infectious. NETs can bind to platelets and red blood cells and impair the coagulation cascade by increasing the efficiency of fibrin aggregation and the risk of thrombotic events. Recently, new information emerged showing that multiple receptors and various redundant signaling pathways are involved in NETosis, suggesting that a fine tuning is strictly required to address different biological effects [106]. Further studies, however, are warranted to thoroughly understand all the different functions of NETs and their importance in the clinical setting.

In light of the pathophysiological relevance of NETs, the development of therapies blocking NETs is ongoing [107]. For example, the inhibition of PAD4 was proved effective in different conditions, such as AMI, stroke, and diabetes [108]. Besides, Janus Kinase (JAK)1/2 inhibitor, that lowers NET formation in mice and humans, was found to experimentally reduce thrombosis [109]. The negative side of these potential therapeutic pathways relies on the non-uniqueness of the targets acting also in processes other than NETosis, hence this research field needs to be further deepened. Along with pharmacological inhibition, some cells (i.e., macrophages) as well as plasma own a system for NET degradation to directly digest extracellular DNA and dampen inflammation, as witnessed by the decreased clearance of NETs occurring in or causing SLE and rheumatoid arthritis [110].

Although the discovery of NETs has revolutionized the understanding of the pathophysiology and the natural history of some human diseases, additional efforts are needed to understand the real impact of NETs and therefore address targeted therapeutic strategies. Finally, a standardized nomenclature and standardized techniques for NET assessment would definitely help to obtain a wealth of comparable data, no matter what the studied disease is [111].

## Figures and Tables

**Table 1:** Main studies investigating NETs in coronary artery disease.

Author	Year	Patients	Biomarkers	Results
Borissoff et al. [29]	2013	282 patients with suspected CAD undergoing coronary CTA, grouped based on the presence and severity of CAD	dsDNA, nucleosomes, citH4, and MPO-DNA complexes	dsDNA, nucleosome, and MPO-DNA complex levels were higher in patients with severe CAD (*p* < 0.05 for all) compared to healthy controls and correlated with the severity of luminal stenosis and the number of diseased coronary artery vessels (*p* ≤ 0.001 for all). Baseline higher-than-median values of dsDNA (OR 3.12, *p* = 0.013), nucleosome (OR 2.59, *p* = 0.030), and MPO–DNA complexes (OR 3.53, *p* = 0.009) were significantly associated with the occurrence of MACEs.
Cui et al. [30]	2013	137 ACS patients (51 UA, 37 NSTEMI, and 49 STEMI), 13 stable AP patients, and 60 healthy controls	dsDNA	ACS patients showed higher dsDNA levels compared to stable AP patients and control group (*p* < 0.05 for both). Significant differences in dsDNA concentrations were observed among UA, NSTEMI, and STEMI sub-groups (*p* < 0.05 for all).
Mangold et al. [28]	2015	111 patients with STEMI undergoing PCI (TIMI flow 0–1)	Nucleosomes and dsDNA	NE, MPO, nucleosome, and dsDNA concentrations were increased at the CLS compared to the femoral site (*p* < 0.001 for all). Nucleosome and dsDNA levels positively correlated with coronary thrombus NET burden (*p* < 0.05 for both), the latter being positively correlated with ST resolution and both enzymatic (CK-MB AUC) and CMR-assessed infarct size (*p* < 0.01 for all).
Helseth et al. [31]	2016	30 patients with CAD undergoing PCI (20 with STEMI and 10 with stable AP)	Nucleosomes and dsDNA	dsDNA and nucleosome levels were higher in patients with STEMI compared to those with AP (*p* < 0.05 for both). dsDNA significantly correlated with peak TnT and CK-MB at day 5 (*p* = 0.03 for both) and with CMR-assessed infarct size at days 5 and 7 (*p* < 0.05 for both), while only nucleosomes correlated with infarct size at day 5 (*p* = 0.02).
Hofbauer et al. [32]	2019	50 patients with STEMI undergoing PCI (TIMI flow 0)	dsDNA and citH3	dsDNA and citH3 levels were significantly increased at the CLS than at the femoral artery (*p* < 0.01 for both). This trend was confirmed only for dsDNA when compared to healthy controls (*p* < 0.0001). Both dsDNA and citH3 were positively correlated with enzymatic infarct size (*p* < 0.05 for both). dsDNA measured at the CLS at the time of PCI was positively correlated with WMSI at the 24 ± 8-month follow-up (*p* = 0.039).
Liu et al. [33]	2019	83 patients with STEMI undergoing PCI (TIMI 0)	dsDNA and MPO-DNA complexes	A larger number of NETting neutrophils from IRA was found compared to peripheral arteries and healthy controls (*p* < 0.05 for both). Higher concentrations of dsDNA and MPO-DNA complexes were retrieved within IRA compared to peripheral arteries (*p* < 0.05 for both). Baseline levels of coronary dsDNA were higher in patients experiencing a MACE (0.70 vs. 0.46 μg/mL, *p* = 0.002). Additionally, dsDNA was found to independently predict in-hospital MACEs (OR 46.26, *p* = 0.001). A cutoff of 0.39 μg/mL for dsDNA was reported as a better prognostic marker compared to TnT and CK-MB (sensitivity 78%, specificity 53%).
Helseth et al. [34]	2019	224 patients with STEMI undergoing PCI followed for 3 months	dsDNA and MPO-DNA complexes	dsDNA and MPO-DNA levels were correlated to leukocyte count at admission (*p* < 0.01 for both) and to each other only in the acute phase (*p* < 0.001), but not after 3 months. dsDNA weakly correlated with glucose in the acute phase and after 3 months (*p* < 0.05 for both), while MPO-DNA did not.
Mangold et al. [35]	2019	91 patients with STEMI receiving thrombectomy during PCI	dsDNA and citH3	dsDNA and citH3 were significantly elevated at the CLS compared to femoral plasma (*p* < 0.0001 for both) and correlated with enzymatic infarct size (CK-MB AUC, *p* < 0.001 and *p* < 0.01, respectively). High CLS dsDNA correlated with low, non-classical (anti-inflammatory) monocyte percentage at the culprit site (*p* < 0.05). Low CX3CR1 expression of non-classical monocytes (i.e., anti-inflammatory) negatively correlated with high CLS dsDNA and citH3 levels (*p* < 0.05 for both).
Liberale et al. [36]	2019	66 patients undergoing PCI	MPO-DNA and TF-DNA complexes	MPO-DNA complexes were higher in the high- compared to the low-CRP group (*p* < 0.01). Patients with high CRP levels showed increased levels of TF-DNA complexes than patients with low CRP levels (*p* < 0.01). A positive correlation with NETosis markers (MPO-DNA and TF-DNA complexes) was recorded (*p* < 0.0001).

ACS: acute coronary syndrome. AMI: acute myocardial infarction. AP: angina pectoris. AUC: area under the curve. citH3: citrullinated histone H3. citH4: citrullinated histone H4. CAD: coronary artery disease. CK: creatine-phosphokinase. CK-MB: creatine-phosphokinase isoform muscle and brain. CLS: culprit lesion site. CMR: cardiac magnetic resonance. CRP: C-reactive protein. CTA: computed tomography angiography. CX3CR1: C-X3-C motif chemokine receptor 1. dsDNA: double-stranded deoxyribonucleic acid. IRA: infarct-related artery. MACEs: major cardiovascular events. MPO/DNA: myeloperoxidase/deoxyribonucleic acid. NET: neutrophil extracellular trap. NSTEMI: non ST elevation myocardial infarction. OR: odds ratio. PCI: percutaneous coronary intervention. STEMI: ST elevation myocardial infarction. TF: tissue factor. TIMI: Thrombolysis In Myocardial Infarction. TnT: troponin T. UA: unstable angina. WMSI: wall motion score index.
